# Configuring a Context-Aware Middleware for Wireless Sensor Networks

**DOI:** 10.3390/s120708544

**Published:** 2012-06-25

**Authors:** Nadia Gámez, Javier Cubo, Lidia Fuentes, Ernesto Pimentel

**Affiliations:** Department of Computer Science, University of Málaga, Campus de Teatinos, 29071, Málaga, Spain; E-Mails: lff@lcc.uma.es (L.F.); ernesto@lcc.uma.es (E.P.)

**Keywords:** context-aware, WSN, middleware, model-driven, configuration, AmI, AAL

## Abstract

In the Future Internet, applications based on Wireless Sensor Networks will have to support reconfiguration with minimum human intervention, depending on dynamic context changes in their environment. These situations create a need for building these applications as adaptive software and including techniques that allow the context acquisition and decisions about adaptation. However, contexts use to be made up of complex information acquired from heterogeneous devices and user characteristics, making them difficult to manage. So, instead of building context-aware applications from scratch, we propose to use FamiWare, a family of middleware for Ambient Intelligence specifically designed to be aware of contexts in sensor and smartphone devices. It provides both, several monitoring services to acquire contexts from devices and users, and a context-awareness service to analyze and detect context changes. However, the current version of FamiWare does not allow the automatic incorporation related to the management of new contexts into the FamiWare family. To overcome this shortcoming, in this work, we first present how to model the context using a metamodel to define the contexts that must to be taken into account in an instantiation of FamiWare for a certain Ambient Intelligence system. Then, to configure a new context-aware version of FamiWare and to generate code ready-to-install within heterogeneous devices, we define a mapping that automatically transforms metamodel elements defining contexts into elements of the FamiWare family, and we also use the FamiWare configuration process to customize the new context-aware variant. Finally, we evaluate the benefits of our process, and we analyze both that the new version of the middleware works as expected and that it manages the contexts in an efficient way.

## 1. Introduction

The usage of Wireless Sensor Network (WSN) technology is nowadays increasing because there are more and more ubiquitous applications that benefit from it. Traditionally, sensor devices have been used to monitor and analyze information about the environment, such as in agriculture [1] or earthquake detection [2]. However, currently they are being used for more dynamic systems, such as those with the purpose of helping humans in daily tasks in Ambient Intelligence (AmI) environments [3,4]. Typical AmI systems are Ambient Assisted Living (AAL; http://www.aal-europe.eu) applications, which provide user-dependent services (such as position location and movement tracking) for elderly and disabled people to enhance their quality of life in their own home by increasing their autonomy and self-confidence. Emergency or unusual situations inducing changes may appear dynamically in AmI applications. Therefore, they have to be capable of handling those changing situations, commonly called *context changes*, detected by means of sensors. Usually, this context information is very complex, because it is made up of data composition coming from variable sensors or other devices. For example, a context related to the daily habits of a user could be composed of a lot of information, such as his/her presence in different areas of the house at certain times, and the frequency and intensity of movement. To gather contexts, these systems should allow cooperation among heterogeneous sensors or devices, which may even represent the same information using different kinds of data (e.g., different units for temperature). Thus, although WSNs provide a very rich set of information, current proposals to gather and analyze this heterogeneous information in applications are tedious and require significant implementation efforts. The development of WSNs software today is tackled by a code-and-fix process that relies solely on the primitive constructs provided by the operating system and the developer skills, without much consideration for maintenance and reuse [5]. Therefore, it would be ideal to be able to provide application developers with a programming abstraction based on methodologies and techniques promoted by software engineering to specify and manage context information for improving the development process and fostering the effectiveness of WSNs in the Future Internet Society.

Context-aware computing [6] is a paradigm of software engineering that covers all the topics related to the building of systems which are sensitive to their context (location, identity, time and activity) by adapting their behavior at runtime according to the changing conditions of the environment, device states, and user preferences or privileges [7]. Thus, context-awareness is the ability to detect and handle context changes. The importance of context-awareness has been recognized beyond its original scope of pervasive and ubiquitous computing [8]. Specifically, context information plays an important role in systems with the technology of WSNs, such as AmI systems, to control the reaction of systems depending on certain situations, to find people with similar interests, and so on. Then, it is essential to manage context information in systems using sensors by reducing human effort in human-computer interaction to make decisions and adapt to a changing environment. However, current programming technology offers only very weak support for developing context-aware applications, and new research is urgently needed to develop innovative Context-Oriented Programming (COP) mechanisms [9].

On the one hand, recent research efforts have tackled the context management in WSNs at middleware level instead of application level [10–17] in order to provide reusable solutions for different applications. However, AmI applications are composed of different heterogeneous devices, and although many context situations can be similar (as movement detection in the smarthomes), these contexts could not be obtained in the same way. For instance, due to the structure of a house, an application can consider seven movement detectors while another one only considers four detectors. Thus, these kinds of applications have a common core asset and a variable part, so it can greatly benefit from Software Product Lines (SPLs) engineering [18], since it is specifically focused on variability modelling. Then, we propose to use FamiWare [19], a family of context-aware middleware built using SPL. In such a way, both complex contexts acquired by heterogeneous devices and the communication of context information among those devices of the AmI system are handled by FamiWare in a transparent way.

On the other hand, we propose to use models to specify the context information for WSNs by using the UML common language. Specifically, we base on the ContextUML metamodel [20], an UML-based modeling language for model-driven context-aware services development, which provides a flexible design of context-aware services. It separates the modeling of context and context-awareness from service components by making easier both development and maintenance of these services.

Then, in this work we present a model-driven process to build context-aware applications based on FamiWare. The contexts for these applications will be specified using ContextUML, and by means of the defined mapping between ContextUML and FamiWare a new augmented version of the FamiWare family with the incorporation of new contexts will be automatically created. Then, taking as input the specific requirements of the system about every device, the network (e.g., number of devices) and the necessities of the application (e.g., security) we will obtain automatically the FamiWare code ready-to-install for every device of the system.

Therefore, the main contributions of our work are the following:

- We take advantage of separating the modeling of context and context-awareness in order to: (i) specify contexts using the ContextUML metamodel; and (ii) make a mapping that automatically transforms the ContextUML elements into elements of FamiWare.
- We define a common architecture easy to reuse for the monitoring and the context-awareness services of FamiWare. We implement these services for three different devices of the middleware family. Concretely, two kinds of sensor devices (MicaZ with TinyOS, and Sun SPOT) and Android-based smartphones and tablets.
- We design a model-driven configuration process that automatically incorporate new contexts to the FamiWare family and generate context-aware versions of the middleware for every application.
- We generate automatically the code of customized versions of context-aware FamiWare for the three different platforms previously mentioned.

The remainder of the paper is organized as follows: Section 2 motivates our proposal presenting the main problems to be solved and how our approach tackles them. In Section 3, we describe the context acquisition and analysis processes in FamiWare. In Section 4, the mapping from ContextUML to FamiWare is defined and illustrated with an AAL case study. Section 5 presents both the implementation of the monitoring and context-aware services in the augmented version of FamiWare, and the code generation process. In Section 6, the evaluation of our approach is detailed. Section 7 compares our approach to related works. Finally, Section 8 outlines some concluding remarks.

## 2. Overview of our Approach

This section motivates and describes an overview of our proposal for managing the heterogeneity of contexts and sensors, and the configuring process of the context-aware middleware for WSNs.

### 2.1. Problem Statement: Context and Sensor Heterogeneity

Context and context-awareness in computing emerged as a part of the ongoing research in ubiquitous or pervasive environments. Dey and Abowd [21] provided the following definition for context-awareness: “*A system is context-aware if it uses context to provide relevant information and/or services to the user, where relevancy depends on the user's task*”. This definition allows developers of context-aware services to define a relevant context for a given scenario, the information which can be used to describe a situation of an entity in an interaction with the system (e.g., user's location to know the temperature of a specific room). Context information is generated by heterogeneous and resource-constrained devices (as sensors). One of the main characteristics of context information is its dynamism, *i.e.*, a context may change at runtime and therefore may require an adaptation of the system. This information needs to be described in a structured and extensible model. In addition, to manage the context, the following functions are required in any context-aware system:

- **Context acquisition.** This task obtains information from different sources. In this work, we focus on the different sensing units integrated into sensor nodes (e.g., acoustic or light sensing units), and both built-in-sensors (e.g., accelerometer) and embedded components (e.g., GPS receiver) into smartphones or tablets. Context acquisition is far from trivial, since normally the contexts are not composed by a single concept coming from a single user or device; instead they use to be made up of several user characteristics and/or environmental information gathered by several devices.
- **Context storage.** In some situations it may be necessary to store some previous values gathered in context acquisition. A local context can be stored locally within the device that acquired it previously. Complex contexts containing information of several sources belonging to the global system must be stored in a centralized node, such as a sink or a server database.
- **Context analysis.** The values collected in the context acquisition function are continuously analyzed and combined to detect a possible context change. The analysis of the heterogeneous contexts gathered previously is a complex task. For example, a context (e.g., temperature) coming from different sensor devices may be given in distinct units (e.g., Celsius or Fahrenheit).
- **Context actuation.** Once a context change is detected, some actions must be executed to adapt the system to the new context situation. These actions can correspond to dynamically reconfigure both the software and hardware involved in the system.

**Figure 1.**
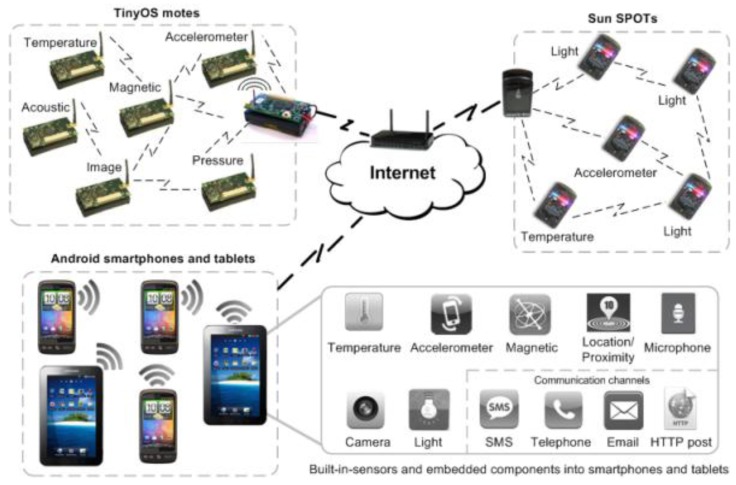
Architecture of a heterogeneous system made up of sensors and smartphones/tablets.

We assume our AmI systems will present an architecture similar to that depicted in Figure 1, where different devices work together to allow the context acquisition and detection of changing situations. Figure 1 shows diverse TinyOS motes and Sun SPOTs with sensing units. Furthermore, smartphones and tablets with Android OS use their components to acquire contexts. These three kinds of devices are communicated via Internet to analyze context data. To manage and exchange contexts efficiently, the four functions described previously can be specified by means of modeling context. Context models define and store context data in a machine readable and processable format. There are several approaches to represent context, as summarized in [22], such as *key-value models* [23], *metamodeling languages* [20,24], *graphical models* [25], or *ontology-based models* [26]. We use a *metamodeling language*, specifically ContextUML to model our context-aware systems. In addition, as FamiWare is built using SPLs and it also uses Feature Models (FMs) [27] to characterize the inherent variability of the AmI domain, including the variable context information, we propose a mapping between the ContextUML elements and the FamiWare Feature Model. This mapping is part of a model-driven process to customize a context-aware FamiWare version as we will see in the next subsection.

### 2.2. Configuration Process of a Context-Aware Middleware for WSNs

**Figure 2.**
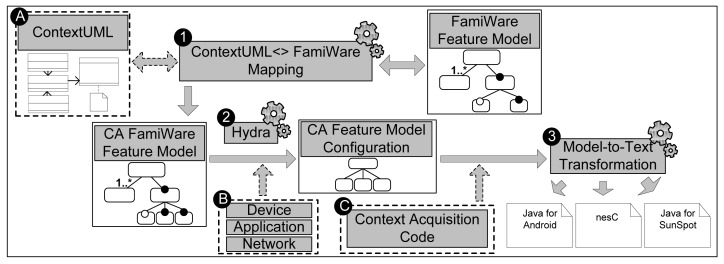
Overview of our approach.

Figure 2 shows our model-driven process to instantiate a context-awareness version of FamiWare for a specific AmI system. The boxes with dashed lines (Figure 2, labels A, B and C) represent the inputs that the AmI application developer must provide to the process. This process is based on the FamiWare Feature Model that specifies which elements of the family are common and which ones are variable independently of the core asset, and enables reasoning about all the different possible valid configurations FamiWare [28]. To customize a context-aware version of FamiWare, we must select which context managed by FamiWare we want to instantiate and determine whether new contexts have to be incorporated to the family. To do this, the application developer must define the contexts to be taken into account in the AmI system using the ContextUML profile (Figure 2, label A). Then, by means of the mapping that we will define in further detail in Section 4 (Figure 2, label 1), an augmented context-awareness version of FamiWare feature model with the new context is automatically obtained. Then, following the steps of the FamiWare customization process, from the augmented FamiWare feature model and the specific requirements of the system (Figure 2, label B) our feature modeling tool (Hydra; http://caosd.lcc.uma.es/spl/hydra) (Figure 2, label 2) generates a specific configuration of FamiWare that deals with these requirements and manages the context specified previously. The system requirements refer to the number or kind of devices, the particularities of the network composed for these devices, and the application necessities with respect to the services that require from FamiWare for every device. These requirements must be specified by the application developer in a form provided by FamiWare. Finally, with this configuration, the FamiWare model to text transformation (Figure 2, label 3) may generate the code for every device of the system (the three different platforms for which FamiWare is currently implemented). To generate all the code, in case FamiWare have to manage new contexts, then the code that indicates how to monitor these new contexts must be implemented and given as input to the configuration process by the application developer (Figure 2, label C). For instance, a presence monitoring algorithm for the cameras that so far is not implemented by FamiWare. After this configuration process, the context-aware FamiWare version that performs the four functions, described in Section 2.1, is generated. Next section will show how FamiWare addresses the functions.

## 3. Context Acquisition and Analysis Using FamiWare

Our solution will address the problems raised during the context acquisition, storage, analysis and actuation by applying different mechanisms, which are detailed as follows:

- **Context acquisition.** To perform this task, FamiWare has a common structure for the monitoring services. It has already implemented several monitoring services (such as location monitoring in mobile sensors or smartphones). In Section 4, we will describe how to add other new monitoring services to the FamiWare family.
- **Context storage.** FamiWare performs this task by keeping context values to be used later. For example, in the TinyOS version, local context data are stored in the flash memory of the sensor.
- **Context analysis.** FamiWare provides a context-awareness service that receives the monitored data to analyze them with the purpose of detecting context changes. For analyzing new context information, which has not been considered in FamiWare previously, we need to add it to the FamiWare family, as we will detail further, in Section 4.
- **Context actuation.** As a context change happens, first the context-awareness service takes the decision of what to perform to adapt the system, and then the FamiWare reconfiguring service is in charge of executing a list of planned actions (e.g., activate a camera or turn off some sensors). Because of the complexity of the reconfiguration process to be performed in distributed systems composed by tiny devices, this task is beyond the scope of this paper. Nevertheless, the interested reader can find further details on how it is addressed by FamiWare in [29].

In order to acquire the context, FamiWare monitoring services have a common structure with the following characteristics: (1) they read data from a sensing unit (e.g., light or temperature) or from other resources to obtain relevant context information (e.g., battery); (2) this reading is done periodically for each specific interval of time or frequency; and (3) the context data is sent to the device in charge of analyzing the context. In a similar way, the context-awareness service implements an interface that allows the definition of data to be under observation as part of the context. For example, a particular instantiation of this service may observe the temperature but not the position. For each context, there is an associated function, which is responsible for detecting context changes that trigger a reconfiguration (e.g., the battery level is under 20%). Once this happens, this service will choose a reconfiguration plan to perform the self-adaptation to adapt the system to the new context situation.

Nevertheless, the number of context situations that may provoke a change in a system is difficult to limit. In this work, we define the model-driven process and the service architecture of FamiWare in order to help application developers to define new contexts in an intuitive way.

**Figure 3.**
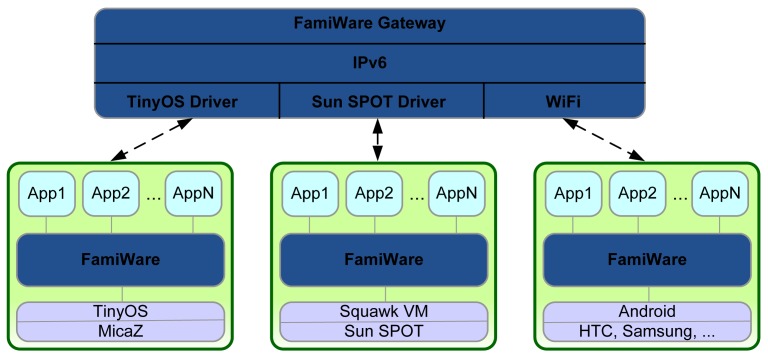
Device interconnection using FamiWare.

In addition, other advantage of using FamiWare is its base infrastructure to hide heterogeneity problems of the communication of context data through the network. Figure 3 shows the communication between the different kinds of devices (the three devices shown in the system of Figure 1) by means of FamiWare. In the figure, three products of the FamiWare family are represented: the version developed in nesC for devices with TinyOS operative system, the Java version for Sun SPOTs that works with the Virtual Machine Squawk VM and the Java version for Android devices. Specific products of FamiWare are installed in each device of a particular system. FamiWare also provides a *gateway* to allow the communication between the heterogeneous devices of the system to deliver the context data from one device to the others and work together transparently.

Currently, in FamiWare several monitoring services are defined and implemented and it considers diverse contexts, such as being aware of the battery level, the location of a device, the light sensed, *etc.* Furthermore, reconfiguration services, which perform the necessary tasks to self-adapt the system, have been implemented in FamiWare. Nevertheless, a certain AmI application may require the management of other context information not considered in FamiWare. In order to reuse the context-aware platform provided by FamiWare, it would be desirable that FamiWare allows the incorporation of new contexts.

Then, if the application developer is interested in managing new contexts, both these contexts and their corresponding monitoring, must be added to the FamiWare family. However, if the AmI application developer is not familiar with feature models, SPLs, and the specific structure of the FamiWare feature model, then adding new contexts could be a difficult and error-prone task. Therefore, we propose a model-driven process to add new contexts automatically to FamiWare and to configure a particular instantiation of the middleware ready-to-install in all the devices involved in the AmI system. As we use a metamodel language, the developer can model contexts by using a common and standard language like UML instead of the tedious task of modifying directly the FamiWare feature model. To achieve this goal, as aforementioned, we define a mapping between the ContextUML metamodel and all the corresponding elements related to contexts in FamiWare. FamiWare feature model represents all the possible context information and changes.

**Figure 4.**
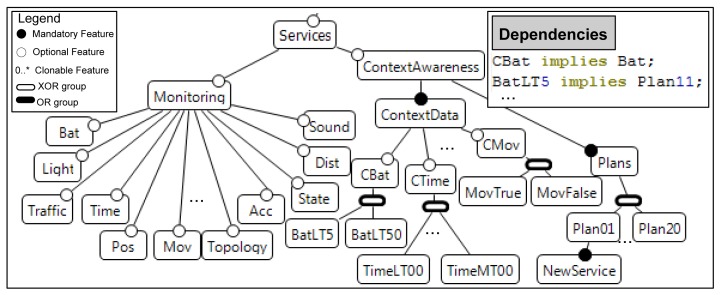
Partial FamiWare feature model of monitoring and context-awareness services.

As shown in Figure 4, in this feature model we model the monitoring services that are dynamically observing the context, the context values which could be considered as context changes, and the corresponding plans to adapt the system to new contexts.

Then, context elements to perform dynamic adaptation in FamiWare are collected by the monitoring services. For example, for the sensor devices we could have the following: battery, traffic, position, topology, state, movement, and so on. Hence, other monitoring services can also be considered in FamiWare, not only for devices, but also for users or environment, such as: user profile, current time, temperature, *etc.* These monitoring services are represented as optional features in the feature model (see Figure 4). Furthermore, if needed, new monitoring services must be added as new optional features to the family. Then, different configurations in each device of an AmI system include only the required monitoring services for the application to avoid the wasting of unnecessary resources.

Context-Awareness service (Figure 4) is responsible for the awareness of the context, using the data provided by the monitoring services, and initiates the reconfiguration process as a consequence of a context change. It is also defined as part of the feature model, since the list of monitored data used for context awareness is also variable. For example, in a specific device configuration of a certain AmI system, it may be useful to consider all its monitoring services observing the context, but in other configurations only the data about the position is considered to perform a reconfiguration. Figure 4 shows that this service is defined by the list of context data (optional) and the plan that must be chosen by this service when a context changes. We define a child feature for each context data, which specifies simple logical expressions that may trigger a context change, as *BatLT5* (battery level less than 5%). To express that some reconfiguration tasks must be performed when the battery level is less than 5%, we must define a cross-tree constraint in the feature model, as is shown in the Dependencies in Figure 4: *BatLT5 implies Plan11*. This means that when the battery level of a sensor is under 5%, then *Plan11* must be executed. We find that as the features in a feature model do not have types, then the way to assign the corresponding values to the features is by means of assigning constraints formatted in plain text (*BatLT5*, equivalent to *Bat* < *5*). So, we enforce that all the constraints that may provoke a context change in FamiWare must be specified as “Monitoring *operator* Value”.

## 4. Handling Context in FamiWare

In this section, we define the mapping from the ContextUML classes and types to the FamiWare elements to add new contexts to the family. We illustrate this mapping with an AAL case study.

### 4.1. Mapping ContextUML to FamiWare

Here, we describe the ContextUML metamodel elements [20] that we use in the mapping with FamiWare. In ContextUML, *Context* is a class that models the context information. This type is divided into two categories formalized by the subtypes *AtomicContext* (low-level contexts that do not rely on other contexts, e.g., patient's location, if we consider a patient as user) and *CompositeContext* (high-level contexts that aggregate multiple contexts, either atomic or composite, e.g., frequency of movement detection in a certain location).

The type *ContextSource* of ContextUML models the resources from which contexts are retrieved. As we advanced previously, we assume AmI systems with diversity of context providers (e.g., sensors, GPS, user preferences, and so on). The ContextUML metamodel provides two context source subtypes: *ContextService* (provided by an autonomous organization, e.g., a glucose sensor), and *ContextServiceCommunity* (that aggregates multiple context services in a unified interface, e.g., WhereAmI to refer the user's location).

The class *CAMechanism* formalizes mechanisms for context-awareness. ContextUML differentiates between two categories of context-awareness mechanisms by subtypes: *ContextBinding* (automatic binding modeling of contexts to context-aware objects) and *ContextTriggering*. *ContextTriggering* models the situation of context adaptation based on context information. For instance, when a user is in a sudden emergency situation, some services or devices of the AmI system must be reconfigured. A context triggering mechanism contains two parts: a set of context constraints, *ContextConstraint* (e.g., a sudden and rapid movement of the user followed by a non-detection of movement may imply that the user has fallen), and a set of actions, *Action* (e.g., search for the nearest sensor with camera, turn on the camera, record a video, and transmit it through the smartphone to the health center). Those actions must be executed if and only if all the context constraints are evaluated to true.

Now we specify the mapping between ContextUML and the elements of FamiWare described in Section 3. Table 1 presents both the correspondences from ContextUML elements (for those elements with any correspondence) to the FamiWare feature model, and their implementation in the middleware.

The *AtomicContext* class in the ContextUML metamodel corresponds to the children of the ContextData feature in the FamiWare feature model (Figure 4). FamiWare Context-Awareness service implements this correspondence by subscribing to the corresponding FamiWare Monitoring service specified by the *ContextService*. The *ContextService* class corresponds to the children of the Monitoring feature and it is implemented by a monitoring service.

**Figure 5.**
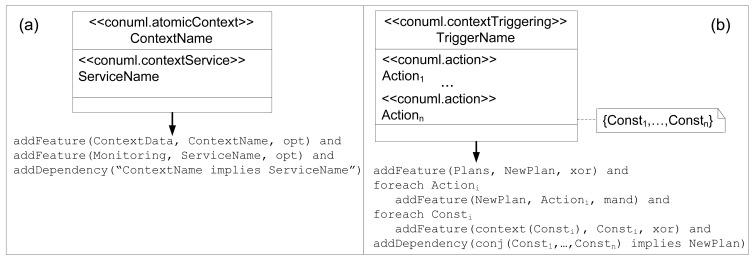
ContextUML to FamiWare mapping.

In FamiWare, which follows a publish/subscribe architecture, each monitoring service publishes a topic with the value of the monitored data and the context-Awareness service subscribed to those that have to be checked to detect possible context changes. At feature model level, as is shown in Figure 5(a), for every atomic context in a model, *i.e., AtomicContext*, we have to add a child optional feature of ContextData. Note that the *addFeature (father, child, variability)* function adds the child feature to the father one (with the assigned variability: optional, mandatory and so on) only when this feature did not previously exist. As is shown in Figure 5(a), we have also to add the corresponding monitoring service as an optional child of the monitoring feature, with the name of the *ContextService*. Finally, a constraint between features must be considered (*addDependency* function), which means that for every ContextData feature that exits its corresponding Monitoring feature is added to the feature model (e.g., *CMov implies Mov*). This means that if we want that a sensor will be aware of the movement, the monitoring service reading the data provided by a movement sensing unit must be instantiated.

As regards the *CompositeContext* subtype, any element is added to the family, since all the atomic contexts that constitute the composite one have been previously added. If a context change is provoked by several context values, this must be specified in the dependencies between features using the *ContextTriggering* (e.g., *MovFalse and TimeMT10*, any movement is detected at more than 10 a.m.).

The context constraint set (*i.e., ContextConstraints* of *ContextTriggering*) are those that trigger a context change. This is specified in FamiWare through a feature model cross-tree constraint between features that indicate that if all the constraints are evaluated to true, this implies that a specific plan will be chosen to perform the system reconfiguration. Also, these constraints (formatted in plain text) must be added as children in their corresponding ContextData feature. The set of actions corresponds with the tasks of a reconfiguration plan used by FamiWare to reconfigure the system [29]. We force that these actions have to be defined in a specific format in ContextUML: *nameoftask(parameters)* in order to make a direct correspondence between ContextUML actions and plan tasks. These tasks are also specified in the feature model as children features of a plan. Then, as is shown in Figure 5(b), for every *ContextTriggering* we add a new plan feature as a child of the *xor*-group of the Plans feature (see in Figure 4). Then, for each *Action* of a *ContextTriggering* we add a new child feature to the new plan. Similarly, for each *ContextConstraint* we add a child feature to the corresponding context *xor*-group. In addition, we add one dependency composed by the conjunction of all the constraints that imply the new plan. All these steps will be illustrated in case study described in the next subsection.

### 4.2. Putting into Practice the Mapping of Contexts in an AAL Case Study

**Figure 6.**
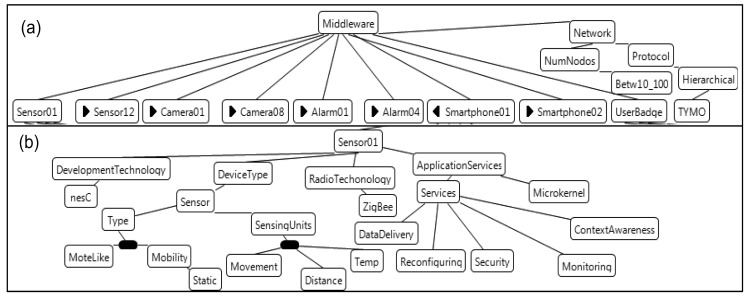
(**a**) AAL feature model configuration; (**b**) Features of a sensor device.

Our case study consists of an AAL home equipped with sensors, smartphones, alarms, cameras, and other devices. User necessities or the structure of the house are different for each AAL home, but both the tasks to be performed and the devices used are similar, especially the tasks related to the context management and the self-adaptation. Then, these systems may take advantage of the benefits of using the context-aware facilities provided by FamiWare. Requirements for every AAL home (including the context to be managed) must be specified, and we will automatically obtain the configuration for each device of the home. Let us consider the home in our example is equipped with 12 sensors, eight cameras, four alarms, and two smartphones. Figure 6 represents an excerpt of the FamiWare feature model configuration for this home using Hydra. Figure 6(a) represents all devices of this configuration, and Figure 6(b) depicts the configuration of a specific device (a sensor). For the sake of simplicity, Figure 6 does not show all the devices and features.

The home in this example has video surveillance capabilities to periodically transmit video to the health center through cameras. The user can communicate directly with the health center through smartphones. Also, an automatic control of the lights and heat, and the handling of alarms are both mechanisms provided. Furthermore, all data transmitted must be encrypted (this is the reason why a Security service is included, Figure 6(b)). This configuration has 12 basic sensors. For instance, *Sensor01* (Figure 6(b)) is a static TinyOS mote sensor whose development technology is nesC and radio technology is ZigBee. This sensor has movement, temperature, and distance sensing units. It provides several services to monitor the environment, to be aware of the context, to reconfigure the system, to deliver and to encrypt data. Similarly, the rest of the sensors have the same or other sensing units, such as light, sound, *etc.*, and they provide some of the previous services. The four alarms and eight cameras are also TinyOS motes, but equipped with actuators and cameras, respectively. The two smartphones have the Android operating system, and they transmit the video received from the camera to the health center, as well as video-conferences. The UserBadge is a Sun SPOT sensor equipped with an accelerometer sensing unit to check the movements of a person.

All these devices are initially active and perform monitoring tasks periodically. However, in some situations it may be more efficient whether some of them are inactive to save energy, in case the user is not going to use some areas of the house. Furthermore, specific services, such as facilities for emerging special situations or a critical development in their illness, may be required. Finally, some services previously running may be considered a waste of resources in this new specific situation, so they must be removed. This means that the system must be reconfigured to adapt to the new context situations.

Let us suppose the AAL home developer wants to consider a new composite context situation which will occur when the user changes his/her daily habits. In this new situation, the user will sleep more hours, he/she will spend more time in his/her bedroom, and the outside lights will be turned off for longer, and so on. These context changes may indicate deterioration in their health. Therefore, a new periodic and automatic video-conference service, by means of a smartphone, with the doctor or the carer must be installed in the system. Furthermore, the bedroom camera frequency for sending images to the health center must be incremented so that the patient can be watched over more efficiently. Finally, to save on resources, sensors or cameras outside the bedroom, which were monitoring or recording periodically, may be in sleep mode or may reduce their monitoring frequency.

To take advantage of the benefits of reusing the FamiWare platform, the new composite context situation will be incorporated to the family. The application developer will model this context using ContextUML, and through our mapping the new context will be automatically added to FamiWare.

**Figure 7.**
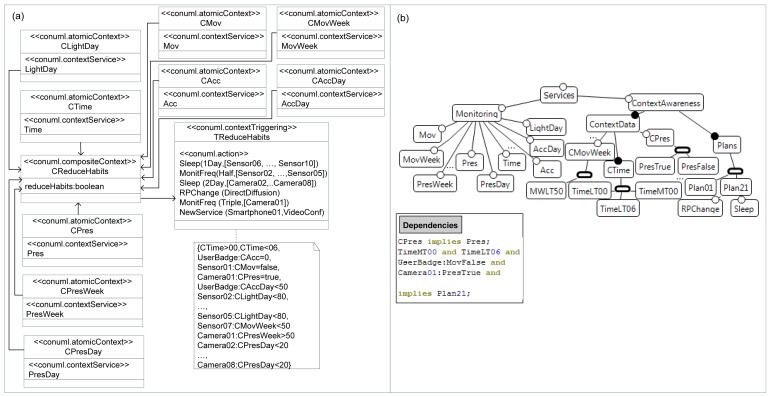
Modeling contexts in our AAL scenario.

Figure 7(a) shows the definition of this composite context (*CReduceHabits*). This context is made up of several atomic contexts, each one with its own context source service: *CTime* (current time), *CLightDay* (hours with light collected by a sensor along a day), *CAcc* (accelerometer angle of the user badge) and *CAccDay* (number of position changes, detected by the accelerometer, accumulated in a day), *CMov* (movement detection) and *CMovWeek* (amount of movement detected by a sensor over the last week), and *CPres* (capability of the camera to detect the presence of a person), *CPresWeek* and *CPresDay* (time of person presence detection for a week or a day, respectively).

The composite context also has context triggering that contains the actions to be executed when some constraints are satisfied. In our scenario, the constraints (Figure 7(a)) are considered to be true if: the current time is between “*00h*” and “*06h*”, the user badge position is horizontal (*i.e.*, the patient is lying down), the bedroom's sensor does not detect any movement, and the bedroom's camera detects the presence of a person in the room, and all these constraints detect whether the user is sleeping in the bedroom at that moment. If this happens, constraints for both the day before and weekly habits are evaluated. These constraints are the following: (1) the movement frequency of the user badge accelerometer is less than 50% with respect to the usual movement; (2) the daily hours with light detected and different sensors are below 80% of the usual hours; (3) the sensor placed just outside the bedroom detects less than 50% of the usual movements over the last week (*i.e.*, user leaving the bedroom has fallen); (4) the bedroom camera detects the patient's presence in the bedroom as more than normal in a week; and (5) the rest of the cameras detect less presence frequency than the day before. When this happens some actions must be performed to reconfigure the system. Firstly, in order to save resources such as energy, some sensors (those on the floor that not correspond to the bedroom) must be in sleep mode, the frequency of the monitoring services (related to the sensors on the floor where the bedroom is) must be reduced, and the cameras located outside the bedroom must also be in sleep. Secondly, as many devices will not be sending data, the network will be extremely reduced, so the routing protocol must be exchanged for another more efficient one for a few devices, like Direct Diffusion. Finally, to control the patient correctly, the frequency of sending data from the bedroom's camera to the health center must be tripled, and a new video-conference service must be installed in the *Smartphone01*. All these elements are automatically mapped into the FamiWare feature model, by increasing the family. For every new atomic context (with its corresponding context service) that previously did not exist in the feature model, we add a new context feature, a new monitor feature, and a usage dependency between them, as seen in Figure 7(b). As FamiWare does not take into account a presence monitoring algorithm for the cameras, the *CPres* and *Pres* features, together with the dependency (*CPres implies Pres*) are included. The same process happens for other new contexts which did not previously exist in the feature model, such as *CAccDay, CMovWeek*, and so on. Therefore, all the context data values that appear in the constraints and were not contemplated in the feature model must be added under their corresponding *xor*-group (e.g., *TimeLT06 or PresTrue*).

As we have previously explained, first we transform each constraint into plain text. Then, a new plan corresponding to the reconfiguration tasks to be executed is created (*Plan21* in Figure 7(b)). The actions will be added as features in the feature model (*Sleep, MonitFreq, NewService*, and *RPChange*). Specifically, these actions will be mapped as a reconfiguration plan, which will be interpreted by the reconfiguration service in order to execute the task (as aforementioned, this is beyond the scope of this paper). The last step of our mapping consists of creating a usage dependency between the constraints and the new plan, as shown in the dependencies, in Figure 7(b).

Throughout this section we have shown how, by taking a simple UML model with the context definition, our family automatically increases with new features and constraints, and several monitoring and context-awareness services previously implemented in FamiWare are reused.

## 5. FamiWare Monitoring and Context-Awareness Services

This section details the architecture, the implementation and the code generation of FamiWare services that manage the context for the three different platforms tackled in FamiWare.

### 5.1. FamiWare Services Architecture and Implementation

**Figure 8.**
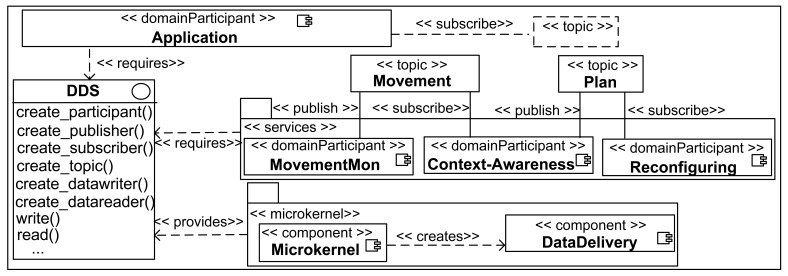
FamiWare autonomic services.

FamiWare follows a publish/subscribe event-based mechanism, specifically a reduced implementation of the OMG Data Distribution Service (DDS) interface [30]. Thus, all FamiWare services communicate with each other by means of this interface that is implemented by a microkernel, as shown in the FamiWare architecture in Figure 8. In this figure, three services related to the context management are represented. The movement monitoring publishes an event (called topic in DDS terminology) with the detection of movement information. The context-awareness service is subscribed to the topics published by the monitoring services. Furthermore, the context-awareness service publishes the adequate reconfiguration plan to adapt the system when a context change is detected. Finally, the reconfiguring service is subscribed to this plan. This is the generic architecture of FamiWare where the specific services also have a common structure to facilitate their reusing. For instance, in the monitoring services the data to monitor (*i.e.*, the context source), the frequency of monitoring and the topic name are generic parameters.

Therefore, for every specific monitoring service FamiWare implements a class for the Java-based implementations (Android and Sun SPOT) or a component for the nesC version that is inherited from an abstract monitoring service. The code of these classes and components and also the corresponding code of the context-awareness services are generated automatically by our configuration process, as will be described in the next subsection.

**Figure 9.**
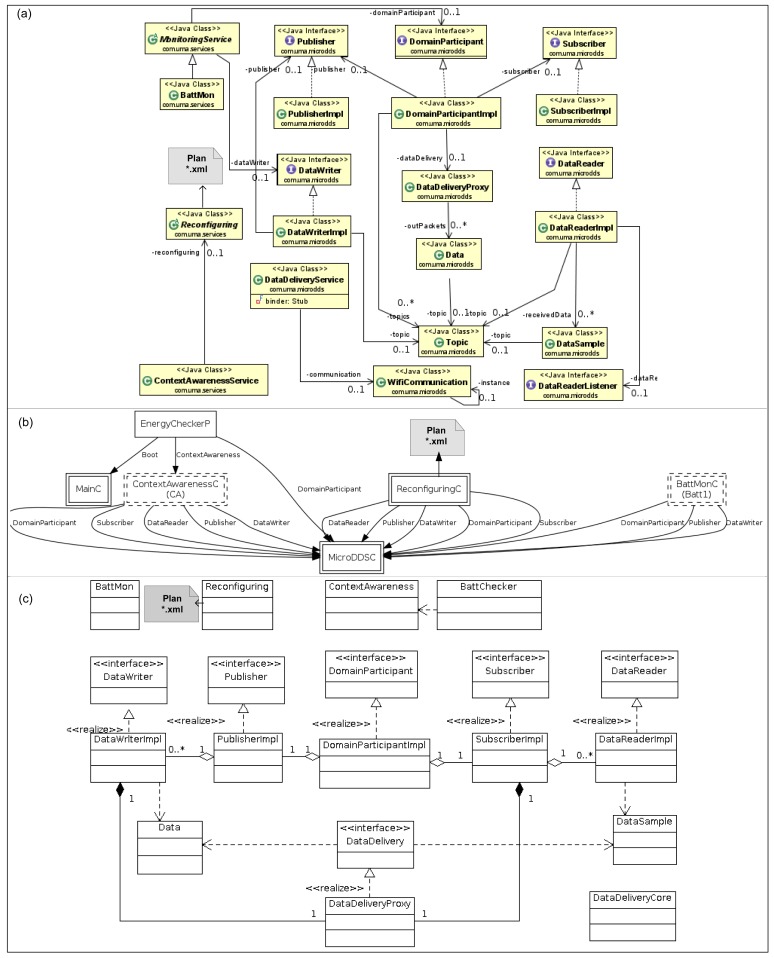
FamiWare instantiations with monitoring and context-awareness services for three platforms: (**a**) Android; (**b**) TinyOS; and (**c**) Sun SPOT.

In the Android version of FamiWare, the services are implemented as an Android *Service* class. The abstract class *MonitoringService* (see Figure 9(a)) has the following characteristics: (i) it reads data from a sensing unit or from other resource considered relevant for the context; (ii) the reading is done periodically; and (iii) the data is published under a determined topic. Then, the kind of data, the reading frequency and the topic name, are parameters that must be obtained after the configuration process. Figure 9(a) depicts the *BatMon* class that inherits *MonitoringService* and implements the reading of the battery level, and also specifies both the reading frequency and the topic name (e.g., *BATT_TOPIC*). The *ContextAwarenessService* defines an interface that allows the specification of new topics to be under observation as part of the context. For example, a particular instantiation of this service could observe the battery level of a sensor and the state of its network connection but not the position of the mote sensor. The context data to be observed and the situations considered as context changes (*i.e.*, the context constraints) must also be obtained after the configuration process. Figure 9(b) shows the component graph for an instantiation of FamiWare with one service to monitor the battery, the context-awareness service and the reconfiguration service.

In a similar way as in the Android version, all the monitoring services (shown as *BattMonC* in Figure 9(b)) are defined through their monitored data, the reading frequency and the topic name of the data specified at design-time. The *ContextAwarenessC* is a generic component that has as parameter, monitored data topics to be subscribed. Likewise in Android, this service has a checker function (*EnergyChecker* in Figure 9(b)) to detect context changes. In the Sun SPOT version (Figure 9(c)), the *Monitoring* services are independent MIDlets and there are specific *ContextAwareness* instances for every context topic subscribed and this instance will have an associated specific checker (*BattChecker* in Figure 9(c)).

### 5.2. Code Generation

In order to instantiate the specific monitoring and context-awareness services for a particular AmI system, we use the model-to-text transformations of FamiWare, which produce the code for deploying the specific middleware to each device. In the case of FamiWare, the microkernel and services are pre-implemented and proved previously, so the automatic code generation entails the selection of FamiWare components and some initialization code. So, the process does not generate code from scratch, it reuses the FamiWare components. However, in the scope of this work we have extended this process for adding new code to monitor and to analyze the new contexts (represented in Figure 2, label C). This code, which refers to the data reading from sensing units or other resources (e.g., GPS), must be implemented previously.

**Figure 10.**
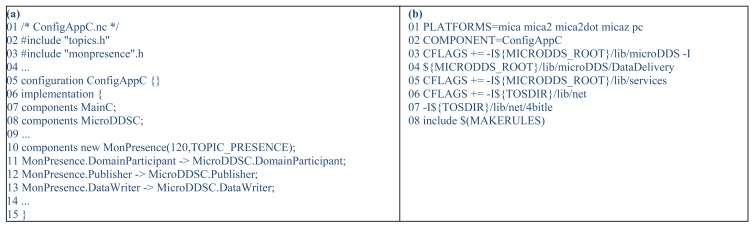
FamiWare code automatically generated for TinyOS sensors.

Since we have designed the architecture of the FamiWare monitoring service in order to be reused easily, then the three generic characteristic defined in the previous section are parametrizable. Then, the application developer must provide the following three parameters: (i) the implementation of the reading function; (ii) the frequency of reading; and (iii) the name of the monitored topic. Therefore, the part of code automatically generated corresponds to the specific middleware configuration indicating which version of which services will be instantiated with which parameters and which topics will be published or subscribed. Our current process generates both code and deployment files for the three platforms described. For TinyOS, it generates the high level configuration file (*.nc) with declarations and connections between the selected services and the makefile used to control the build process of a project. For Android, an Android Activity (*.java) is generated. This activity initiates the selected services by using Android Intents and calls the Android startService() method. Furthermore, our process generates an Android Manifest (*.xml) with the needed middleware information for the Android system. Finally, for Sun SPOT, the process generates a MIDlet (*.java) that initiates the selected services and the Manifest (*.mf) that declares these services. For example, Figure 10 shows an excerpt of the code automatically generated by our process for a TinyOS sensor device. Figure 10(a) shows the configuration nesC file and the associated makefile is shown in Figure 10(b). In the configuration file, firstly the *MainC* mandatory component of TinyOS (line 07) and the FamiWare microkernel (called *MicroDDSC*, line 08) are declared. Then, a component for the new monitoring of presence used in the cameras is firstly created (line 10) and secondly associated to a *DomainParticipant, Publisher* and *DataWriter* with the microkernel (lines 11–13). This configuration file (*ConfigApp.nc*) is called in the Makefile (Figure 10(b), line 2).

## 6. Evaluation

In this section, we discuss the main advantages and drawbacks of our approach, and we evaluate, by means of experimental results, whether the implementation of our approach is feasible.

### 6.1. Benefits of the Reconfiguration Process

As we mentioned in previous sections, WSNs and AmI systems may obtain the benefits of building application families instead of building single applications. This is because these kinds of systems have many common parts and some differences, and the common parts will be reused, specifically the parts related to the handling of contexts. Once a SPL family has been defined and implemented, the instantiation of particular products is a fast and easy task. Nevertheless, the effort needed to define a family from scratch is considerable, so we propose the usage of FamiWare. One of the main advantages of using FamiWare to develop context-aware AmI application families is that they can use the implementation of many AmI typical services (as data delivery and security), as well as the FamiWare facilities for sensors and smartphones. One of these facilities is the provided structure to manage contexts in order to build self-adaptative systems, a strong requirement for WNSs in the Future Internet. Thus, these applications benefit greatly from using the FamiWare services for handling the contexts or adding new contexts to the family by means of our automatic configuration process.

In our case study, for *Sensor01*, 18 components (taking into account the FamiWare microkernel, monitoring and context-aware services, as well as other services) are reused from a total of 24 components. These six new components correspond with the monitoring services to acquire the new contexts. But these components are no implemented from scratch, the only code that must be implemented are the one corresponding to the acquisition functions. Then, the monitoring services are automatically generated. Therefore, for our case study 86% of code lines are automatically generated and only 14% must be implemented by the application developer. In the best case, where all the context considered for a system has been already implemented by FamiWare, 100% of code will be generated automatically and the application developer only must to indicate the requirement about devices, network and application. In the worst case, where all the contexts needed by the AmI system has not been implemented by FamiWare, the application developer will benefit from the communication facilities and other services provided by FamiWare, as well as from the common structure of monitoring and context-awareness services which allows the automatic generation of code. Then, the effort of implementing a new context-aware application will be reduced considerably.

One drawback of our defined mapping is that if the application developer models a context that exists in FamiWare but with a different name, so far our process cannot detect that they refer to the same context. In order to achieve this goal we plan to use OWL-S ontologies, which are well known in the knowledge representation community to model concepts and the relationships that hold between them. Specifically, we will define a formal representation of a set of concepts (such as user, profile, activity, location, time, *etc.*) within a domain by capturing the relationships between those concepts and making the automatic selection of context services possible.

### 6.2. Experimental Results

To evaluate whether our approach is feasible we have installed an instantiation of FamiWare with the new contexts of our case study (detailed in Section 4.2), and we have measured the interval time from which the context-awareness service receives the context information and detects a context change until the reconfiguration is completed. This interval time encompasses the following: the context-awareness service receives the monitored data (via several monitoring services implemented in MicaZ and Sun SPOTs sensors), analyzes them to detect a context change, chooses the suitable plan, and finally the reconfiguration service receives and executes the plan, reconfiguring diverse devices. Table 2 reports these results.

The measurements were taken using the emulator provided in the r07-x86 SDK of the Android version for Linux, and also using the HTC Desire smartphone device. The implementation for the motes has been realized in TinyOS 2.1.1 and the experiments have been performed using two simulators, TOSSIM and AVRORA beta-1.7.106, and also in real MICAz motes (equipped with CC2420 radio and an ATmega128L microcontroller). Finally, for the Sun SPOTs sensors the implementation has been done using the Sun SPOT Development Kit (rev8), yellow-101117-1 (v6.0). The tests have been performed in the Solarium emulator and in Sun SPOTs devices (with CC2420 radio and the microcontroller AT91SAM9G20). The total time of the context-awareness service is 8.22 ms, an insignificant time compared with the time taken by the reconfiguration service until the system is reconfigured, 1756.98 ms. The longest time in the reconfiguration service corresponds to the execution of the tasks that comprise the plan (see Table 2). This entails sending several events to nodes which takes a time highly depending on the routing protocol used, the structure of the network, and the number of nodes. Therefore, the overhead produced to reconfigure the system is insignificant compared with the time of sending a packet. However, from the user point of view, the total time taken to detect changes and reconfigure the system (less than 2 seconds) is satisfactory. We quantify the memory footprint used by FamiWare, since memory constraints are very strong in AmI devices. FamiWare consists of a microkernel and a variable set of services, so the memory usage on a device depends on the actual configuration. For TinyOS, the minimal core platform consists of the microkernel and the data delivery service. Also, since any TinyOS application is compiled together with TinyOS in one image, we also have to include it as part of our measures. Specifically, to be aware of the context, the minimal instantiation must have one monitoring service, the context-awareness service and the reconfiguration service. Considering the RAM memory of MICAz is 4 Kb, this FamiWare version consumes 54.4% (2,229 bytes) of RAM. On the other hand, as average considering 12 monitoring services and 7 other services FamiWare uses 79.9% (3,274 bytes) of the memory, so there is still 20.1% free. The FamiWare project with the context-aware services for the Sun SPOTs version uses as average 298 bytes, that means 29% of the total RAM memory of SunSpot devices, 1 Mb. Finally, for the Android version, the Android processes share a memory region, so the memory footprint of FamiWare remains *constant* although the number of services increases. For a typical configuration for autonomic tasks (with 7 services), the microkernel and a simple application (2,824 Kb), FamiWare uses 1.51% (8,920 Kb) HTC Desire mobile memory (576 Mb), so this percentage goes down to 1.51%. Summarizing, the resource consumption of FamiWare is minimal and therefore very well suited to the resource requirements.

**Figure 11.**
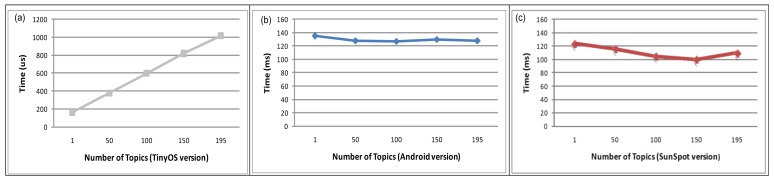
Reception of monitored data when many topics published.

Now, we evaluate the scalability of FamiWare, regarding the delay produced in the reception of the monitored data as the number of monitoring services and topics increase. We have performed some experiments with the hypothesis that there is an overload in the context-awareness side (*i.e.*, the subscriber) when the number of monitored topics increases (*i.e.*, publishers). For the TinyOS version, the subscriber has to search the topic when an event arrives. Since the topics are stored in a table, the time to search a topic lineally increases with an increasing number of topics as shows Figure 11(a). For 195 topics, it takes 1.013 ms so the overload produced is not very significant compared to the time taken to send a packet through the network (e.g., 6.43 s for three intermediate nodes,). However, the tests are not successful with a number of topics larger than 195. This is because the system is out of memory and the stack overflows. Therefore, we can conclude that the boundary in the TinyOS version is 195 monitored topics, more than enough for an AmI system. The time consumption is hard to measure in Android or Sun SPOT versions, since as with any Java-based system, this strongly depends on the state and the timing of the garbage collector. As shown in Figure 11(b,c), increasing the number of topics does not produce overload in the subscriber side for Android and Sun SPOT versions. This is because topics are not stored in a table, as in TinyOS, and instead, a *Topic* object is constructed from the topic name, using the reflective mechanism of Java and is notified to the corresponding *DataReaders*. Then, this time depends more on the notification time to the *DataReaders* than on the number of topics, resulting in very variable times. Comparing the three implementations, the two Java-based versions are more scalable than the TinyOS one.

## 7. Related Work

This section compares our proposal to related work in both middleware solutions for context-awareness in WSNs, and modeling context-awareness focused on model-driven approaches and reconfiguration of AmI systems, in order to show the advantages of our approach.

### 7.1. Middleware Solutions for Context-Awareness in WSNs

*Context Toolkit* [10,11] is one of the first developed context-aware frameworks, and it aims at facilitating the development and deployment of context-aware applications. The Context Toolkit consists of context widgets and a distributed infrastructure that hosts the widgets. Context widgets provide developers the benefits of encapsulating the complexity of the sensors, abstracting context information and providing reusable and customizable building blocks of context sensing. Applications can access context information from context widgets using two methods: (i) a widget provides a set of attributes that can be queried by applications; and (ii) applications can register to be notified of context changes detected by the widget, and the widget trigger callbacks to the application when changes in the environment are detected. FamiWare follows this latter method where applications use monitoring services that subscribe to context-awareness services to reconfigure the system. The main advantage of our approach is that FamiWare implements the context related services for real devices, and especially we proof the feasibility in specific sensors with very limited capabilities as the MICAz sensors.

Context-Awareness Sub-Structure (*CASS*) [12] is server-based middleware intended to support context-aware applications on hand-held and other small mobile computers. A key feature of CASS is its support for high-level context data abstraction and the separation of context based inferences and behaviors form application code. As in our approach, this separation opens the way to make context-aware applications configurable by developers. In CASS, the analysis of the context data is always done in a centralized server. Taking into account that most costly operations in WSNs are the communication tasks, this is not a good solution for systems with a high number of sensors. Then, we propose a mixed solution where the simplest contexts could be analyzed in a distributed way in small sensors, and other more complex contexts could be analyzed in higher capacity devices such as smartphones that punctually could act as a centralized node. The purpose of FamiWare is always to reduce the number of communications for detecting context changes.

*C-CAST* [13] is a context management system based on a consumer-provider broker model, where providers employ a common context representation format, decoupling various entities involved in the production and consumption of context information. All communication in the system is based by exchange of Hypertext Transfer Protocol (HTTP) messages [31] using a Representational State Transfer (REST) interface [32], which allows components to be ported onto almost any networked device. Context providers are responsible for accessing and gathering context information from any kind of sensor. Context consumers may be any kind of application utilizing the context information. The context broker will forward any context changes from the context provider to the subscribed context consumer. This context broker is centralized, and then it has the same limitation as CASS. Furthermore, the system does not provide any simple way to process context information before it reaches context consumers. Instead, FamiWare provides a context-awareness service that analyzes the context information and react when a context change is detected.

*MidSen* [14] is an architecture that bridges the gap between multiple applications running at application level and deployed sensor networks. MidSen has adopted a rule based engine to handle system dynamics, and makes applications flexible by allowing them to update their rules against knowledge base. MidSen middleware makes it easy to design new applications the at application layer without worrying about the details of the underlying deployed sensor network. The goal of this middleware is similar to ours. However, the authors only provide a common architecture and any particular real implementation in device sensors is given. Then, they do not consider some crucial restrictions of these devices, such as the energy consumption. As future work, they plan to incorporate an energy component for effective resource utilization of the network. Compared to our approach, the energy saving is one of the main purpose of FamiWare.

*WiSeKit* [15] is a distributed middleware approach for addressing the dynamicity of WSN applications by enabling adaptation and reconfiguration. As we propose, using this middleware the developer only focuses on application-level requirements for adaptivity, while the underlying middleware services expose off-the-shelf APIs to formalize the process for adaptive WSN application development and hide the complexity of the technical aspects of adaptation in context-aware scenarios. WiSeKit follows the situation-action rules approach defined by the framework presented in [33], which models sensor network context information from different sources, and based on the notion of context node maps their context model to software components, processes the context data for the use of adaptation reasoning service, and implements the context model. Then, similar to FamiWare, it adopts dynamic parameters and component adaptation, and follows an approach based on situation-action rules as our context-awareness services. However, their reconfiguration process is driven by code instead that our driven by model, with the advantage that supposes the management of the context at the model level hiding the complexity of the heterogeneity of devices if it is done at the code level.

*COPAL* [16] is a runtime context provisioning middleware that, via a loosely-coupled and composable architecture, ensures context information from WSNs and other sources can be processed for the needs of context-aware applications. Based on the COPAL architecture and context provisioning models, a Domain Specific Language (DSL) [34], COPAL-DSL, is proposed to facilitate the development of context provisioning plans, by reducing the development efforts of context provisioning using automatic code generation. They distinguish three general types of components in a context provisioning (gathering, transferring and processing context) system: publishers, processors, and listeners. Publishers sense the environment and report their findings and listeners react based on this stimulus. This is equivalent to the monitoring services that we will provide. Processors are situated between the publishers and listeners and they process the stimuli and can infer information about the environment. We will implement a context-awareness service to perform this function. Authors have also defined a macro language, COPAL-ML [35] that extends Java programming language and is tailored for the application development using COPAL in order to reduce the development efforts, and separate concerns of the context-aware application from underlining WSNs. Compared to our proposal, for this purpose, we propose the usage of a common language as UML to avoid the application developers have to know about specific languages.

*Lamses* [17] is a middleware for larger-scale for Ubiquitous Sensor Networks (USNs), considering that USNs have received considerable attention and have been employed in various applications, such as effective energy usage, office administration support, shopping and entertainment, *etc.* In addition, the prevalence of USN computing environments raises the issue of how applications can take full advantage of context-aware information. In this approach, USN middleware is the core of the system, and responsible for the sensor networks management, data storage, data query processing, communication between USN applications and the middleware, and so on. Lamses uses context-awareness-based lightweight data to determine whether the sensed data (from a specific sensor) contains errors and to manage the abnormal situation. It also provides a common network interface for collaboration with sensor sinks in USNs. Compared to our proposal, this approach does not provide a model solution to specify which context must be taken into account.

Summarizing, the main goals of these middlewares are similar to ours. But most of them mainly focus on one specific kind of platform (sensors or mobiles), so none of them offer context-aware middleware solutions that will be configurable in an automatic way for different kind of devices.

### 7.2. Modeling Context-Awareness

Prezerakos *et al.* [24] present an approach with the ability to handle context with minimal user intervention, by decoupling core service logic from context-aware functionality. They take advantage of using ContextUML, as does our approach, but only focused on solving service composition and not in reconfiguration unlike contrary to our proposal, since we provide FamiWare to perform reconfiguration when context changes are detected. In [36], Serral *et al.* present a model-driven development method which allows the automatic generation of context-awareness AmI systems from models. In contrast to our approach, this method does not reconfigure the system automatically according to dynamic context changes. Furthermore, another difference between these two approaches and our work is that we do not only allow the modeling of the context, but also this modeling is part of a model-driven configuration process to build context-aware applications. In [37], Segarra *et al.* describe a multi-level model to build context-aware self-adaptive entities in an AAL application. The model defines both mandatory functionalities (observe, decide, plan, and execute, corresponding with the four steps of FamiWare autonomic computing process) and composition coordination of adaptive entities, which can be customized by adaptation designers. Compared to this proposal, we base ours on a model-driven strategy by achieving a high level of abstraction that allows the separation of modeling context and context-awareness from services, which makes both development and maintenance of context-aware services in the self-adaptation paradigm easier. Similar to our approach, Giner *et al.* [38] propose a methodology based on feature models for defining self-adaptive AAL services by considering dynamic reconfiguration. As we have discussed throughout this paper, we think feature models may be not so intuitive when new contexts are added. Therefore, we propose the use of an UML-based modeling language, to manage contexts, and provide an automatic mapping between ContextUML elements and FamiWare features. Finally, Hefeida *et al.* [39] present a WSN context model in collaborative sensor network applications that captures multiple context parameters in multiple dimensions, (*i.e.*, context from/to different layers of the network stack). This approach is similar to a supply and demand model, where nodes attempt to meet application demands while considering the current node/network state and balancing the load across the network. Thus, this model takes context awareness to a dimension by sharing context information between layers (cross-layer information) and hence resulting in a more informed decision, and also communicates context information between nodes to distribute the load over the network (inter-nodal context sharing). In a similar way, our model also addresses the complex context made up of data composition coming from heterogeneous devices. But, unlike this proposal of modeling context, which is more focus on the communication of context parameters between all layers, our aim is to use models to define the context information for tackling the heterogeneity existing in WSNs by means of a model-driven process which separates the modeling of context and context-awareness from service components, and makes easier both development and maintenance of context-aware applications.

## 8. Conclusions

In this paper, we have presented the main difficulties in the management of complex contexts for systems made up of different kinds of sensors and other devices. In addition, we have argued the use of a middleware to hide this complexity. Instead of using a single middleware, we propose the use of a family of context-aware middlewares, FamiWare, prepared to be installed in the heterogeneous devices that may comprise an AmI system. We have described the model-driven configuration process to add new contexts to the FamiWare family. Our context modeling for FamiWare is based on the ContextUML metamodel. Thus, for adding new contexts the application developer can model how to acquire and analyze the context information using the ContextUML profile. Then, by means of our mapping, from the ContextUML elements to the FamiWare feature model, new contexts are automatically incorporated into FamiWare, augmenting the family to consider these new contexts. After this, our configuration process generates automatically the FamiWare code to be installed in every device of a certain AmI system. Furthermore, we have described the generic architecture of context-aware services in FamiWare, and we have detailed their implementation for three different kinds of devices. Finally, in order to prove that our approach works as expected, we have modeled a new complex context for an AAL case study and we have implemented and reused the corresponding code, thereby testing the feasibility. Currently, we are working on combining our context modeling with the power of representing contexts by means of OWL-S ontologies in order to understand their semantics and recognize contexts related to each heterogeneous stakeholder.

## Figures and Tables

**Table 1.** Correspondences of ContextUML with FamiWare feature model and implementation.

**ContextUML**	**FamiWare Feature Model**	**FamiWare Implementation**
Context **AtomicContext**	ContextData child feature	New subscription of the Context-Awareness service to monitored information
ContextSource **ContextService**	Monitoring child feature	New Monitoring service
CAMechanism ContextTriggering **ContextConstraints**	The conjunction of the constraints is the first element in a usage dependency (*i.e., implies* cross-tree constraint) between features	Constraints to be analyzed in the Context-Awareness service to detect context changes
CAMechanism ContextTriggering **Action**	Children features of a new plan created as a Plan child. It will be the second element in the usage dependency	Tasks of an OWL-s reconfiguration plan. This plan will be executed when the previous constraints are evaluated to true

**Table 2.** Latency for the reconfiguration of the system.

**Operation**	**Time**
Receive monitored data	5.37 ms
Detect a context change and choose the plan	2.85 ms
Total Context-Awareness time	8.22 ms
Receive plan chosen	4.64 ms
Get and read the plan file	15.92 ms
Interpret and execute the tasks of the plan	1736.42 ms
Total Reconfiguration time	1756.98 ms
